# Synthetic Aperture Radar Target Recognition with Feature Fusion Based on a Stacked Autoencoder

**DOI:** 10.3390/s17010192

**Published:** 2017-01-20

**Authors:** Miao Kang, Kefeng Ji, Xiangguang Leng, Xiangwei Xing, Huanxin Zou

**Affiliations:** 1 School of Electronic Science and Engineering, National University of Defense Technology, Sanyi Avenue, Changsha 410073, China; kangmiao15@163.com (M.K.); luckight@163.com (X.L.); hxzou2008@163.com (H.Z.); 2 Beijing Institute of Remote Sensing Information, Beijing 100192, China; xingxiangwei@nudt.edu.cn

**Keywords:** SAR target recognition, feature fusion, stacked autoencoder, MSTAR

## Abstract

Feature extraction is a crucial step for any automatic target recognition process, especially in the interpretation of synthetic aperture radar (SAR) imagery. In order to obtain distinctive features, this paper proposes a feature fusion algorithm for SAR target recognition based on a stacked autoencoder (SAE). The detailed procedure presented in this paper can be summarized as follows: firstly, 23 baseline features and Three-Patch Local Binary Pattern (TPLBP) features are extracted. These features can describe the global and local aspects of the image with less redundancy and more complementarity, providing richer information for feature fusion. Secondly, an effective feature fusion network is designed. Baseline and TPLBP features are cascaded and fed into a SAE. Then, with an unsupervised learning algorithm, the SAE is pre-trained by greedy layer-wise training method. Capable of feature expression, SAE makes the fused features more distinguishable. Finally, the model is fine-tuned by a softmax classifier and applied to the classification of targets. 10-class SAR targets based on Moving and Stationary Target Acquisition and Recognition (MSTAR) dataset got a classification accuracy up to 95.43%, which verifies the effectiveness of the presented algorithm.

## 1. Introduction

The development of synthetic aperture radar (SAR) technology has witnessed the explosive growth of available SAR images. Manual interpretation of numerous SAR images is time-consuming and almost impractical. This has significantly accelerated the development of automatic target recognition (ATR) algorithms. Choosing efficient features is important for traditional SAR ATR techniques and many feature extraction methods have been developed to describe the targets in SAR images [1,2,3,4,5]. In practical applications, however, it is difficult to fully describe target characteristics to achieve a high recognition accuracy with a single feature.

Feature-level fusion of SAR images can not only increase the feature information of the images to perform comprehensive analysis and integration processing, but also can effectively integrate the advantages of various features to reduce the complexity of training and improve algorithm adaptability. Currently, the feature fusion algorithms are mainly divided into three categories [6]: The first category is a method of feature combination, specifically, combining the features in series or in parallel according to certain weights for obtaining a new feature vector [7]. Feature selection, the second category which utilizes a variety of preferred methods, selects an optimum feature combination to obtain a low-dimensional feature showing a better discrimination [8,9]. The third category is feature transformation, which is a way to convert raw features into new feature representations [10,11]. However, due to the difficulties in exploring deep information from raw features, the traditional methods have unsatisfactory performance on redundancy reduction and efficiency improvement.

Recently, feature fusion based on a deep neural networks has performed excellently in various fields [12,13,14], which has prompted scholars to conduct research on SAR imagery target recognition with neural networks [15,16,17]. However, the remarkable performance of a deep neural network requires a large number of tagged data as training samples, which is hard to accomplish in SAR target recognition. In addition, complex SAR images usually require a complicated network structure to fit them. With the increaing complexity, the training time of the network will significantly increase. Thus, under the condition of a relatively small amount of sample labels, simplifying the network structure and enhancing the training efficiency can improve the performance of SAR ATR technology.

Stacked autoencoder (SAE) [18], as a type of unsupervised learning network, converts raw data into more abstract expressions through a simple non-linear model and fuses features by optimization algorithms. The feature integration based on SAE can substantially reduce the redundancy and complement information between the features. With its relatively simple structure, SAE can effectively adapt to the needs of fast SAR image interpretation and achieve stronger generalization capability with less training samples. Many scholars have conducted research on this area. Ni et al. [19] flattened the SAR images into one-dimensional vectors and fed them into SAE after preprocessing and segmentation, and this method obtained a more competitive result. To fit the complex raw data, 64 × 64 neurons are required in the first layer of the SAE, which leads to a complex SAE structure and low network training efficiency. In [20], texture features of SAR images extracted with a Gray Level Co-Occurrence Matrix (GLCM) and Gabor wavelet transform were optimized to a higher-level feature by SAE. However, due to the great redundancy between texture features, the added information is relatively small, and accordingly, the effect of the integration is not obvious. Chen et al. [21] combined the spatial features and spectral information of hyperspectral images with SAE. This achieved a better performance in classification tasks, but the raw spectral information with high dimensions led to a relatively complicated network structure. Additionally, by employing a multi-layer autoencoder, reference [22] extracted contour and shadow features of SAR images and integrated into the synergetic neural network (SNN) to identify the target, which enhances the classification accuracy to some extent. It has to be noted that the algorithm requires segmenting the shaded area of targets and this processing is relatively complex. 

The main goals of this work were to obtain distinctive features by fusing features and to improve the efficiency of recognition by simplifying the network structure of SAE. With an unsupervised learning algorithm, SAE can not only prevent network from overfitting when the number of labeled samples is relatively small, but also effectively provide a deep integration of features with its nonlinear mapping ability. In addition, considering the information redundancy and complementation, 23 baseline features [23] and a local texture feature called Three-Patch Local Binary Pattern (TPLBP) [24] of SAR images are extracted. The selected features will describe SAR images from local and global perspectives, and provide complementary information for the SAE network under the condition of lower feature dimensions. Therefore, the fused features will be more robust and discriminative. 

The remainder of this paper is organized as follows: Section 2 displays the framework of the proposed algorithm. Section 3 describes the details of baseline features and TPLBP features. Section 4 introduces the SAE and the softmax classifier utilized in this paper. Experiments based on MSTAR database [25] are carried out in Section 5 to evaluate the proposed method and compare its performance with other algorithms. Section 6 concludes this paper. 

## 2. Proposed Approach

**Figure 1:**
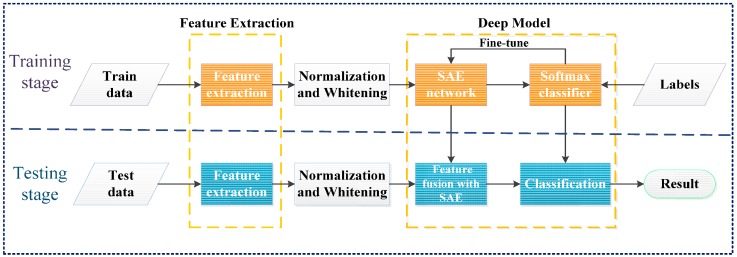
The feature fusion flowchart.

This section introduces the structure of the proposed approach. Similar to most successful and commonly used feature learning models, the procedure of the feature fusion algorithm proposed in this paper is divided into a training stage and a testing stage, presented with the flow chart shown in Figure 1. The proposed algorithm consists of the following steps to learn robust fused features: (1) cut the SAR images into the same size and extract features from these images; (2) subtract the mean value and then apply Zero Component Analysis (ZCA) whitening to pre-process the features; (3) cascade the features and feed them into the SAE to pre-train the network; (4) train the softmax classifier with the fused features and fine-tune the model according to the training data labels. 

(1) Feature Extraction

For a SAR ATR system, feature selection plays an important role in the target recognition. In this paper, features with less redundancy are extracted to simplify the SAE inputs. Firstly, the geometric parameters of the targets are extracted in order to get the baseline features with 88 dimensions. Meanwhile the 128-dimensional texture feature vector, which is obtained by connecting the histogram of TPLBP value in series, combines with the baseline features to form a cascaded feature vector with 216 dimensions. Compared with the deep learning methods which import the whole image into neural network, the proposed approach decreases the dimension of raw data from 16,384 to 216. This greatly reduces the number of neurons in the first layer. The details of the features will be introduced in Section 3.

(2) Normalization and Whitening

In this step, 216-dimensional features are normalized by subtracting the mean value of the feature space and then apply ZCA whitening [26],which is common in deep learning. The purpose of ZCA whitening is to reduce the redundancy between features, and to make all input elements have the same variance. The ZCA whitened data are calculated as ***X**_ZCAWhite_* = *T**X***, where *T* = *UP*^−(1/2)^*U^T^* and *U* and *P* are the eigenvectors and eigenvalues of the covariance matrix of ***X***: $\sum =\frac{1}{m}\sum_{i=1}^{m}(x^{\left(i\right)}){\left(x^{\left(i\right)}\right)}^{T}$.

(3) Deep Model

After being normalized and whitened, the cascaded vectors are imported into the SAE to train the first layer with a gradient descent algorithm which is able to optimize the cost function. The hidden layer provides what it learned as the inputs for the next layer of SAE. Meanwhile, it is necessary to fix the first layer’s weights of the network when the second layer is training, and all of the following layers are supposed to be trained in this way.

The features obtained from the SAE can be applied to classification by feeding the output of the last layer to a classifier. In this paper, a softmax classifier is adopted. According to the distance between labels and the result of classification, the network weights are fine-tuned with a back-propagation algorithm. When the training of the network is completed, the classification performance is evaluated with the extracted feature of test samples. The deep model consists of a SAE and a softmax classifier, which will be described in Section 4.

## 3. Feature Extraction

In this section, 23 kinds of baseline features and local texture features are chosen as the fusion data. Two types of features will be integrated to supply richer information for the SAE to learn. Fisher score [27] is utilized to select the baseline features of SAR images. Moreover, comparing the LBP value of different regions, TPLBP texture features obtain a robust representations of the targets.

### 3.1. Baseline Features

Baseline features [23] are a collection of geometry parameters about SAR target area. For a pixel in a complex-valued SAR image, the position of the pixel is represented with (*a,b*), and it can be expressed as *c*(*m,n*) = *i*(*a,b*) + j*q(*a,b*), where *i*(*a,b*) and *q*(*a,b*) are the real and imaginary parts of the complex-valued SAR image, respectively. Then the following equation can be used to describe the power detection of the pixel’s magnitude:

(1) $$p\left(a,b\right)={\left[i\left(a,b\right)\right]}^{2}+{\left[q\left(a,b\right)\right]}^{2}$$

With the method of an adaptive threshold based on entropy, which is proposed by Kapur et al. [28], a binary image can be obtained. After morphological dilations [29], unconnected region of the image is removed to extract geometry features of the binary image or the dilated image, which form the multi-dimensional baseline features. 

**Figure 2:**
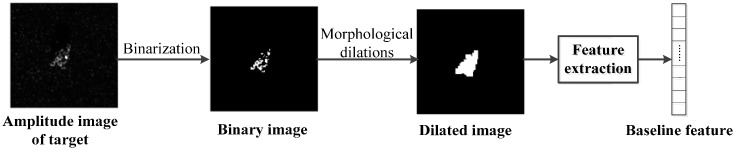
Framework of feature extraction.

This paper selects 23 kinds of geometry features that achieve higher score in feature ranking and obtains an 88-demensional baseline feature vector. The framework of this procedure and the categories of features are shown in Figure 2 and Table 1, respectively.

The details of the features are as follows:

(1) NumConRegion: the number of connected regions in the binary or dilated binary image.
(2) Area: the total number of pixels with value one in the binary or dilated binary image.
(3) Centroid: the center of the mass of the binary or dilated binary image.
(4) BoundingBox: the smallest rectangle containing the mass of the binary or dilated binary image
(5) MajorLength: the length (in pixels) of the major axis of the ellipse that has the same normalized second central moments as the mass of the binary or dilated binary image.
(6) MinorLength: the length (in pixels) of the minor axis of the ellipse that has the same normalized second central moments as the mass of the binary or dilated binary image.
(7) Eccentricity: the eccentricity of the ellipse that has the same second-moments as the mass of the binary or dilated binary image. The eccentricity is the ratio of the distance between the foci of the ellipse and its major axis length. The value is between 0 and 1.
(8) Orientation: the angle (in degrees ranging from −90 to 90 degrees) between the x-axis and the major axis of the ellipse that has the same second-moments as the mass of the binary or dilated binary image.
(9) ConvexHull: the matrix that specifies the smallest convex polygon that can contain the mass of the binary or dilated binary image. Each row of the matrix contains the x- and y-coordinates of one vertex of the polygon. The first row is selected here to construct the feature vector.
(10) ConvexHullNum: the number of the vertices of the smallest convex polygon that can contain the mass of the binary or dilated binary image.
(11) ConvexArea: the number of the pixels in the convex hull that specifies the smallest convex polygon that can contain the mass of the binary or dilated binary image.
(12) FilledArea: the number of pixels with value one in the Filled image, which is a binary image (logical) of the same size as the bounding box of the mass of the binary or dilated binary image, with all holes filled in.
(13) EulerNumber: the number of objects in the mass of the binary or dilated binary image minus the number of holes in those objects.
(14) Extrema: the matrix that specifies the extrema points in the mass of the binary or dilated binary image. Each row of the matrix contains the x- and y-coordinates of one of the points. The format of the vector is [top-left top-right right-top right-bottom bottom-right bottom-left left-bottom left-top].
(15) EquivDiameter: the diameter of a circle with the same area as the mass of the binary or dilated binary image.
(16) Solidity: the proportion of the pixels in the convex hull that are also in the mass of the binary or dilated binary image.
(17) Extent: the ratio of pixels in the mass of the binary or dilated binary image to pixels in the total bounding box.
(18) Perimeter: the distance between each adjoining pair of pixels around the border of the mass of the binary or dilated binary image.
(19) WeightCentroid: the center of the mass of the binary or dilated binary image based on location and intensity value. This measure is also based on the power-detected SAR chip.
(20) MeanIntensity: the mean of all the intensity values in the mass of the power-detected image as defined by the binary image or the dilated binary image.
(21) MinIntensity: the value of the pixel with the lowest intensity in the mass of the power-detected image as defined by the binary image or the dilated binary image.
(22) MaxIntensity: the value of the pixel with the greatest intensity in the mass of the power-detected image as defined by the binary image or the dilated binary image.
(23) SubarrayIndex: the cell-array containing indices of pixels within the bounding box of the binary image or the dilated binary image. The first and last elements of each cell are selected here to construct the features.

**Figure 3:**
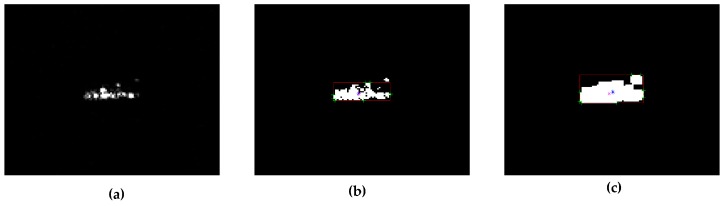
Examples of baseline features. (**a**) A SAR chip processed by energy detection; (**b**) A binary image of SAR; (**c**) A dilated binary image of SAR. In (**b**,**c**), centroid, boundingbox, extreme and center of gravity of target area were marked in blue, red, green and magenta.

Figure 3 shows a portion of the features extracted from BMP2 slices in the MSTAR database. They are SAR chip after power detection, binary image, dilated image and partial features, respectively. Meanwhile, the centroid, boundingbox, extreme and center of gravity of target area were marked in blue, red, green and magenta.

### 3.2. TPLBP Operators

The echo signals of radar waves vary because of the differences in structure, roughness and physical characteristics of a target, while the texture information of SAR targets changes little along with the azimuth of the target. Thus, texture features can be used for target identification. Local Binary Pattern (LBP) is a simple and effective local texture extraction operator. It can effectively use the spatial information and adequately reflect the local spatial correlation of images with gray-scale and rotation invariance. 

The traditional LBP operator is described as follows: within a window sized 3 × 3, compare the gray value of the center pixel with that of the other adjacent eight pixels. If the gray value of adjacent pixels is greater than the central pixel’s, the value of adjacent pixels is marked as 1, otherwise it is marked as 0. In this way, the eight pixels in the neighborhood will produce an unsigned 8-bit number called LBP value, which reflects the texture information of the region. Limited by the size of the neighborhood, LBP operator cannot describe the large-scale texture information, and original LBP operator does not have a rotational invariance. In terms of these aspects, it is not suitable to describe the target in azimuth sensitive SAR images.

**Figure 4:**
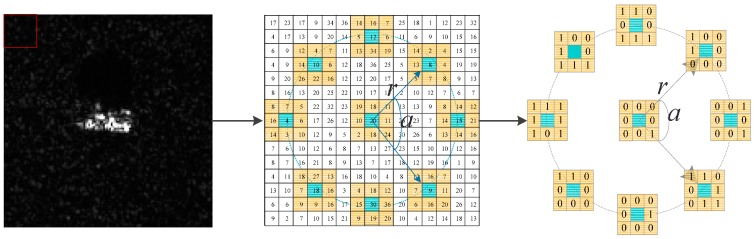
The TPLBP code of BMP2 (S=8,w=3,α=2).

Wolf et al. [24] improved the LBP operator and proposed the Three-Patch Local Binary Pattern (TPLBP). Firstly, for each pixel in the image, considering a w×w patch centered on the pixel, S additional patches distributed uniformly in a ring of radius r around it. Utilizing the LBP operator mentioned above, the center pixel of each patch can obtain the LBP value. Specifically, as is shown in Figure 4, with the parameter set as S=8,w=3,α=2, the LBP value of *S* patches of a certain pixel is produced in an area of the red box which is marked in the SAR image. The values of two patches, which are α-patches apart along the circle, are compared with those of the central patch and their similarity is calculated further.

Then applying the following equation to each pixel, the TP LBP value can be calculated:

(2) $$TPLBP_{r,s,\omega ,\alpha }\left(p\right)=\sum_{i}f(d(C_{i}{-C}_{p})-d(C_{i+\alpha \mathrm{mod}s},C_{p}))2^{i}$$

where $C_{i}$ and $C_{i+\alpha \mathrm{mod}s}$ are pairs of patches along the ring and $C_{p}$ is the central patch. The function d(⋅,⋅) is a certain distance function between two patches and f is defined in Equation (3), where τ is set to 0.01 [24,30]:

(3) $$f(x)=\{\begin{array}{cc} 1, & x\geq \tau \\ 0, & x<\tau \end{array}$$

After the TPLBP value of every pixel in the image is obtained, the whole image is divided into non-overlapping rectangular patches of equal size (*B* × *B*). After the frequency for TPLBP value of each rectangular patch is calculated, the histogram vector of each rectangular window will be connected in series in order to form TPLBP feature vector. 

Compared with LBP, the TPLBP operator suppresses the speckle noise more effectively. It contrasts the LBP value of patches, which describes the relationship between adjacent patches, rather than the gray value between pixels. In addition, selecting the patches in a circle allows the features to have rotation invariance. Furthermore, the parameters *r* enables TPLBP to compare different texture features of various scale, which overcomes the shortcomings of LBP with limited range and effectively describes texture features in large-scale SAR images.

## 4. Deep Model

Our deep model will be trained and validated with labeled samples in the training stage. Then in the testing stage, it will be fixed to evaluate the performance of network by the testing data. In this section, a brief introduction of a SAE and a softmax classifier is given.

### 4.1. Stacked Autoencoder

**Figure 5:**
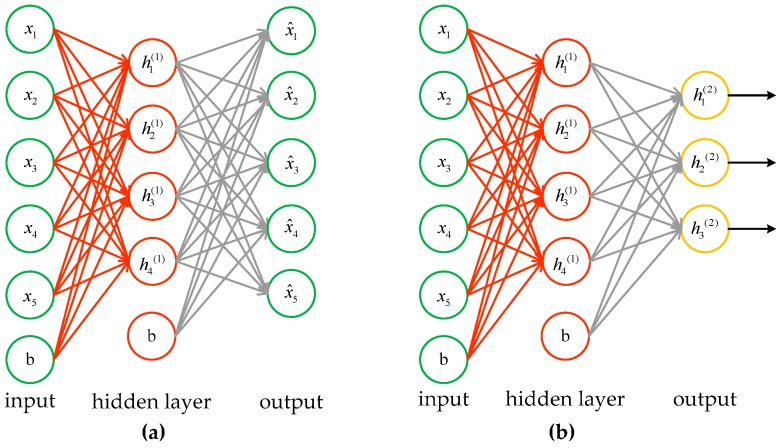
The structure of autoencoder and SAE. (**a**) A three-layers autoencoder; (**b**) A SAE composed of two autoencoders.

Autoencoder [18] is usually composed of three layers, as shown in Figure 5a. The encoder, which consists of an input layer and a hidden layer, converts an input signal **x** to **a**. Likewise, the hidden layer and output layer constitute a decoder in order to transform **a** to output signal $\hat{x}$. It can be expressed as follows:

(4) a=f(Wx+b)

(5) $$\hat{x}=g(\hat{W}a+\hat{b})$$

where **W** and $\hat{W}$ are the weight matrixes of encoder and decoder, respectively. Additionally, f(⋅) and g(⋅) are the mapping functions such as sigmod function or tanh function. When $\hat{x}\approx x$, it is considered that the autoencoder reconstructs the input. For the dataset containing *m* samples, the cost function is defined as follows [20]:

(6) $$J(W,b)=[\frac{1}{m}\sum_{i=1}^{m}\left(\frac{1}{2}{\left\|{\hat{x}}_{i}-x_{i}\right\|}^{2}\right)]+\frac{\lambda }{2}\sum_{l=1}^{n_{l}-1}\sum_{i=1}^{s_{i}}\sum_{j=1}^{s_{i+1}}{\left(W_{ji}{}^{\left(l\right)}\right)}^{2}$$

where $x_{i}$, ${\hat{x}}_{i}$ represent the *i*-th input and the *i*-th output of *l*-th layer, respectively. $W_{ji}{}^{\left(l\right)}$ indicates the connection weights of the *i*-th neurons of layer l with the *j*-th neurons of layer l+1 and b represents the bias term. Furthermore, the first term of Equation (6) is a mean squared error term. The second part is a regularization term, and it can be seen as a way to compromise between small weights and minimized cost function. The relative importance of the two elements is determined by the value of λ.

Generally, a sparse restriction is given on the weight of the network, that is, sparse autoencoder, so as to obtain a better feature representation ability. When $a_{j}{}^{\left(2\right)}$ expresses the output of a hidden neuron from the encoder with a given input ***x***, then equation ${\hat{\rho }}_{j}=\frac{1}{m}\sum_{i=1}^{m}\left[a_{j}{}^{\left(2\right)}(x_{i})\right]$ represents average activation of hidden unit. The average activation ${\hat{\rho }}_{j}$ is set to ρ which is called sparsity parameter and typically has a small value close to 0 [31].

To satisfy the network sparse constraints, an extra penalty term will be added to cost function so that it penalizes ${\hat{\rho }}_{j}$ deviating significantly from ρ. Then the overall cost function expressed as follows [31]:

(7) $$J_{sparse}(W,b)=J(W,b)+\beta \sum_{j=1}^{s_{2}}KL(\rho ,{\hat{\rho }}_{j})$$

(8) $$KL(\rho ,{\hat{\rho }}_{j})=\rho \ln\frac{\rho }{{\hat{\rho }}_{j}}+(1-\rho )\ln\frac{1-\rho }{1-{\hat{\rho }}_{j}}$$

where *KL* represents Kullback-Leibler divergence between a Bernoulli random variable with mean ρ and a Bernoulli random variable with mean ${\hat{\rho }}_{j}$. The weight of sparsity penalty term is determined by β. With backward propagation algorithm, partial derivatives of the cost function can be calculated. In order to solve the optimal model, gradient descent algorithm is used to update parameters W and b.

A SAE is a neural network consisting of multiple layers of sparse autoencoders in which the output of each layer is wired to the inputs of the successive layer, as shown in Figure 5b.

A good way to obtain optimal parameters for a SAE is to use greedy layer-wise training [32]. $W^{\left(k,i\right)}$ represents the weight of the i−th layer in k−th autoencoder. Firstly, parameters $W^{\left(1,1\right)},W^{\left(1,2\right)},b^{\left(1,1\right)},b^{\left(1,2\right)}$ of the first layer can be obtained by training on raw inputs, which transforms the raw inputs into a vector consisting of activation of the hidden units. Secondly, the second layer is trained on this vector to obtain parameters $W^{\left(2,1\right)},W^{\left(2,2\right)},b^{\left(2,1\right)},b^{\left(2,2\right)}$. Finally, repeat the steps above and use the output of each layer as input for the subsequent layer to complete the pre- training. One of the characteristics of this approach is to freeze the parameters of the rest layers of the model while training a certain layer. After the pre-training is accomplished, fine-tuning with back propagation [33] can be applied to improve the results.

Instead of random initialization of parameters, with an unsupervised pre-training process, SAE initializes the parameters to easily convergent values. This method indicates the contents of the hidden layer what to learn. Additionally, the introduction of sparsity prevents network overfitting and improves generalization ability of the network. The process of fine-tuning uses global supervision so that the network converges to global minimum. Consequently, SAE allows learning the deep features of inputs with powerful feature representation capacity.

### 4.2. Softmax Classifier

The softmax classifier is a promoted logistic regression classifier, which can effectively cope with multiple classification problems. In order to improve the performance, this paper fine-tunes the SAE with a softmax classifier by calculating the probability represented by reference [34]:

(9) $$p(y_{i}=j|x_{i};\theta )=\frac{e^{\theta_{j}{}^{T}x_{i}}}{\sum_{l=1}^{k}e^{\theta_{j}{}^{T}x_{i}}}$$

where $\theta_{j}$ is the parameter vector and as usual an iterative optimization algorithm such as gradient descent will be utilized to minimize the cost function of classifier. After obtaining optimal model parameters, the samples are classified to the highest probability category.

## 5. Experiments

To verify the validity of the proposed algorithm, the following experiments were designed. Firstly, in order to determine the structure of the SAE and achieve the best target recognition performance the influence of network structure on classification accuracy was investigated by changing the number of neurons in the hidden layers of the SAE. Subsequently, the distribution of features is visualized, which contributes to figuring out what the SAE did in the fusion procedure. The comparison of classification accuracy between raw features and fused features demonstrates the effectiveness of feature fusion. Compared with other algorithms, the SAR feature fusion algorithm based on SAE is demonstrated to have better performance on recognition performance and efficiency.

**Figure 6:**
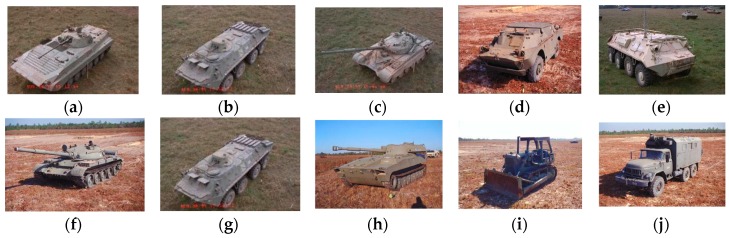
The optical images of ten military targets in MSTAR dataset. (**a**) BMP2-C21; (**b**) BTR70; (**c**) T72-132; (**d**) BRDM2; (**e**) BTR60; (**f**) T62; (**g**) ZSU234; (**h**) 2S1; (**i**) D7; (**j**) ZIL131.

The experiments are conducted on the MSTAR dataset for 10-class targets recognition (armored personnel carrier: BMP-2, BRDM-2, BTR-60, and BTR-70; tank: T-62, T-72; rocket launcher: 2S1; air defense unit: ZSU-234; truck: ZIL-131; bulldozer: D7). In order to comprehensively assess the performance, this paper chooses SAR images of 17° and 15° aspect as training samples and test samples, respectively. Details are displayed in Figure 6 and Table 2.

Experimental parameters: according to reference [23], the parameters of the TPLBP operator are set as S=8,w=3,α=1,r=12,τ=0.01,B=64 in order to extract the 128-dimensional TPLBP feature. Then it is combined with 88-dimensional baseline features in series. After importing the feature vectors into a SAE, the softmax classifier is employed for target recognition.

### 5.1. The Influence of the SAE Network Structure on Performance

In the training stage it is easy for a neural network to get trapped into “overfitting” and “underfitting” problems. With a certain scale of training data, the probability of overfitting will rise with the increase of the neurons. Thus, it would be advisable to keep the number of layers as less as possible on the premise of accuracy. Therefore, a SAE with two hidden layers for feature fusion is adopted to explore the effect of neurons number on generalization capacity.

In order to correctly configure the SAE network, 20% of the training samples (17°) were randomly selected as the validation set, and the remaining were used as the training set. The training set is used to adjust the parameters of SAE network, and the best model is selected according to the accuracy on the validation set. Finally, the performance of the model is tested on the testing set (15°).

Geng et al. [20] pointed out that in order to prevent the network from overfitting or underfitting, the number of hidden layer neurons should not be too small nor too large. Thus, in this paper, $L_{1}$ and $L_{2}$ represent the neural number of the first hidden layer and the second hidden layer, respectively, where $L_{1}\subset [100,700]$ and $L_{2}\subset [100,700]$. Weight decay parameter λ controls the relative importance of the mean squared error term and weight decay of cost function as mentioned earlier. In this paper, a small λ ranging from $e^{-6}$ to $e^{-3}$ is adopted, so that the mean squared error term accounts for more proportion of cost function than weight decay. Furthermore, sparsity parameter is usually a small value approximating to 0, leading to better generalization ability in the network. Parameter β controls the weight of sparsity penalty factor, and the parameters are set as follows $\rho =\;0.1,\;\beta \;=\;3,\;\lambda \;=\;5e^{-4}$.

**Figure 7:**
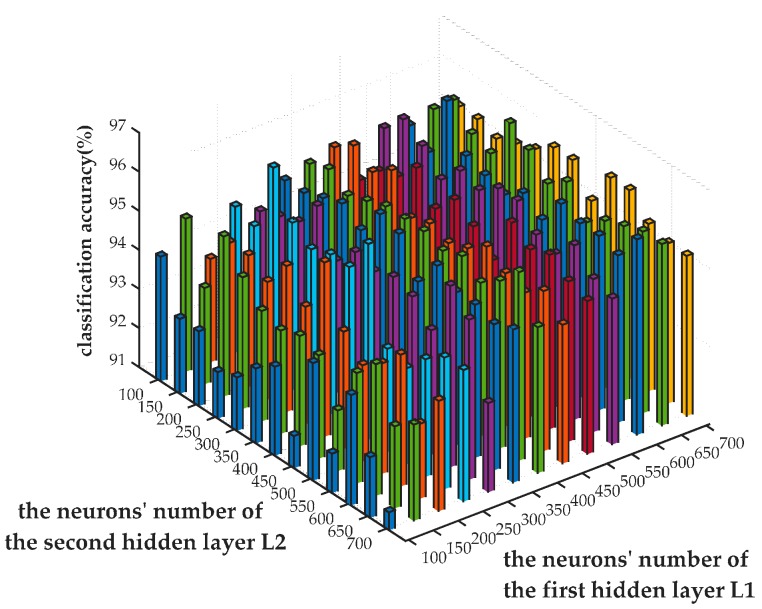
The effect of changed neuron’s numbers on classification accuracy.

Next, under the condition of the same parameters and inputs, the classification accuracy on validation set with changed number of neurons is recorded. To ensure the precision of the results, the experiment was executed five times under each group of parameters, and the mean value on each case was calculated. The comparison results are shown in Figure 7. 

Figure 7 shows that the number of neurons in each layer has a significant impact on network performance. When $L_{2}$ is fixed, the classification accuracy varied along with the change of $L_{1}$. Best performance was achieved while the value of $L_{1}$ approximates to 600. Similarly, the value of $L_{2}$ is supposed to be set to 200 for higher accuracy with a fixed value of $L_{1}$. Given $L_{1}=600,L_{2}=200$, the network obtained the highest recognition accuracy on validation set, amounting to 96.36%. Therefore $L_{1}=600,L_{2}=200$ is considered the best SAE configuration and it is tested with the testing set independently, obtaining a classification accuracy of 95.43%. 

As can be seen from the figure, the accuracy is relatively higher when $L_{1}$ is larger than $L_{2}$. It is possible that the input data can be precisely fitted when the number of neurons in the first hidden layer is larger than the second one’s, which decreases the reconstruction error in the first hidden layer. Conversely, if the number of neurons in the first hidden layer is relatively small, the reconstruction error of the input data will accumulate in the network, degrading the network performance. However, with limited samples, the classification accuracy will not increase unlimitedly since the parameters of network model will grow rapidly with the increase of hidden neurons. This leads to more freedom of network parameters and probably causes over-fitting.

### 5.2. The Comparison of Different Features

**Figure 8:**
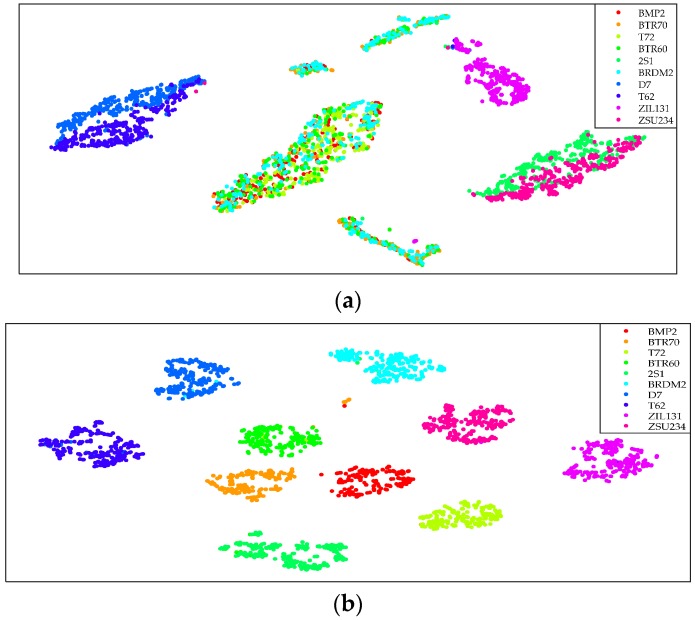
The distribution of cascaded features and fused features. (**a**) The distribution of cascaded features; (**b**) The distribution of the fused features in the same feature space.

In this experiment, 88-dimensional baseline features and 128-dimensional TPLBP features were extracted from SAR images at 17° in MSTAR collection, and then they were compared with the fusion features. Utilizing t-distributed stochastic neighbor embedding proposed in reference [35], the cascaded features and fusion features were visualized so as to obtain the distribution in a two-dimensional space. The results are shown in Figure 8.

As shown in Figure 8, in two-dimensional space, the distribution of cascaded features obviously presents interclass overlap and intraclass dispersion. However, the fusion features of 10-class targets are separated independently. Therefore, in the process of feature fusion, SAE learns more useful information from the input data and fuses the features effectively by changing the spatial distribution of input data.

Next, baseline features and TPLBP features are provided as training data for SAE, respectively, and their recognition performance is compared with fused features, in which SAE network has two hidden layers. The structure and parameters of network are adjusted to achieve the best outcomes. The results are recorded in Table 3.

**Figure 9:**
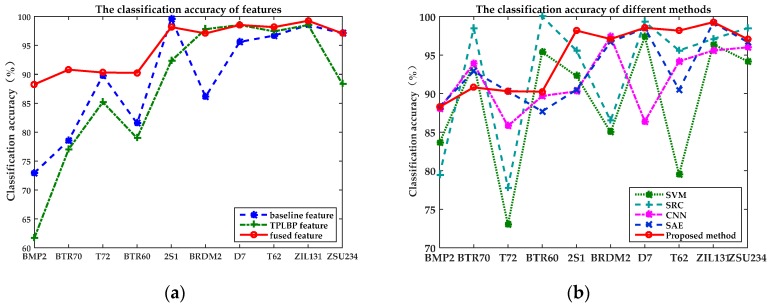
The classification accuracy of 10-class targets. (**a**) Comparison of the classification accuracy of baseline features, TPLBP features and fused features on 10-class targets; (**b**) Comparison of the classification accuracy of different method including SVM, SRC, CNN, SAE and the proposed method.

Table 3 shows that the classification accuracies of baseline features and TPLBP features are 90.81% and 89.19%, respectively. The classification accuracy of fusion features is up to 95.43%, increasing by almost 5%. Additionally, the baseline features have a relatively lower classification performance on the BRDM2, while the TPLBP features show better discrimination in this category. After the feature fusion, the classification accuracy of BRDM2 is up to 97.08%. The same situation occurs to ZSU234. It is shown that the proposed method integrates the complementary information in the raw features, thus making up the shortage of a single kind of features. It is found that the recognition rate of BMP2, BTR60 and BTR70 is relatively low. Correspondingly, the distribution of those targets is near in the feature space. The reason is that all of the three categories belong to armored personnel carriers. And they have some similarities on shapes and structural characteristics, which increases the difficulty in distinguishing. In addition, Figure 9a shows that the fused features have a similarity trend with baseline features and TPLBP features in classification accuracy. This indicates that the selection of raw features has a direct impact on the fusion results. Moreover, the higher accuracy on the most of categories of fused features reveals that SAE is able to extract more distinguishable features from the raw features. After representation conversion with SAE, fused features is more robust and distinctive.

In order to verify the advantages of the fused features on recognition, this paper employs Support Vector Machines (SVM) for classification with the baseline features, TPLBP features, cascaded features and fused features, respectively. Similar to the proposed method, the training set (17°) mentioned in Table 2 are applied to training the SVM, and the optimal parameters are obtained by 5-fold cross-validation. After the model was determined, its performance is evaluated on the testing set (15°). As is shown in Table 4, the classification accuracy of fused features is higher than other features, which demonstrates that the proposed method effectively integrates the information of features to improve the discrimination of features.

### 5.3. Comparisons with Other Methods

Table 5 shows the classification accuracy of different algorithms. In reference [36], two typical classification approaches, Sparse Representation-based Classification (SRC) and SVM, were applied for 10-class targets recognition on MSTAR dataset, obtaining the classification accuracy 86.73% and 89.76%, respectively. Reference [37] proposed a convolutional neural network (CNN) to extract features from 128 × 128 SAR images to train a softmax classifier, in which the classification accuracy with the same experiment settings was up to 92.3%, which it is lower than the accuracy achieved by the proposed algorithm. A comparison of classification accuracy of 10-class targets was plotted in Figure 9b, which shows that the proposed algorithm has better classification accuracy than the other algorithms’ in six target categories. Although the other four categories are slightly lower than with the other algorithms, the classification accuracy is still acceptable.

To display the advantages of the feature fusion algorithm on time complexity and classification accuracy, after pre-processing with ZCA whitening, the 128 × 128 SAR images were flattened into one-dimensional vectors and were directly fed into a SAE comprised of two hidden layers. The classification accuracy obtained from the SAE is 93.61%, which is lower than the proposed algorithm. Table 6 shows the 10-class targets confusion matrices of the SAE trained on images and the proposed method. Compared with the SAE trained on images, the proposed method achieved the same accuracy on BPM2 and T72 and higher accuracy on the rest of categories except for BTR70. As is shown on Table 3, the baseline features and TPLBP features both have a relative low accuracy classification on BTR70. Therefore, they provide less discriminative information for SAE to fuse, which leads to a poor performance of proposed method.

As shown in Table 7, the training time and testing time of the two algorithms mentioned above were compared. Experiments are implemented with Matlab R2014a on a computer equipped with a 3.4 GHz CPU and 64 G RAM memory. The proposed method is almost 12 times faster than SAE in training time and 72.5 times faster in testing time. Consequently, feature fusion based on SAE can effectively reduce the number of neurons, simplify the network structure and improve the efficiency of algorithms with limited training samples.

## 6. Conclusions

In this paper, we have proposed a feature fusion method based on SAE for SAR automatic target recognition. Baseline features and TPLBP features are fused in a well-designed SAE and further fed to a softmax classifier for recognition. Experiments are conducted to explore the influence of configuration of the SAE used in feature fusion, and a 95.43% classification accuracy is obtained. Utilizing feature visualization, it reveals that the SAE changes the spatial distribution of raw features during the process of feature fusion, which increases the inter-class distance and reduce the intra-class distance. Additionally, the comparison of classification accuracy among different features shows that the baseline features and TPLBP features have good complementarity and the fused features have better discrimination. Compared with other algorithms, the proposed method simplifies the network structure and improves the recognition accuracy and time efficiency. Since the selection of features has a great impact on target recognition, in order to choose the features with more fusion value and further enhance the performance of recognition, we will conduct further studies on the selection of an appropriate feature selection algorithm and the relationships between different features.

## Figures and Tables

**Table 1:** The selected baseline features.

**Number**	**1**	**2**	**3**	**4**	**5**
Feature Name	NumConRegion	Area	Centroid	BoundingBox	MajorLength
**Number**	**6**	**7**	**8**	**9**	**10**
Feature Name	MinorLength	Eccentricity	Orientation	ConvexHull	ConvexHullNum
**Number**	**11**	**12**	**13**	**14**	**15**
Feature Name	ConvexArea	FilledArea	EulerNumber	Extrema	EquivDiameter
**Number**	**16**	**17**	**18**	**19**	**20**
Feature Name	Solidity	Extent	Perimeter	WeightCentroid	MeanIntensity
**Number**	**21**	**22**	**23**		
Feature Name	MinIntensity	MaxIntensity	SubarrayIndex		

**Table 2:** Number of training samples and test samples.

Targets	BMP2	BTR70	T72	BTR60	2S1	BRDM2	D7	T62	ZIL131	ZSU234	Total
17°	233	233	232	256	299	298	299	299	299	299	2747
15°	196	196	196	195	274	274	274	273	274	274	2426

**Table 3:** The classification accuracy of raw features and fused features.

Categories	BMP2	BTR70	T72	BTR60	2S1	BRDM2	D7	T62	ZIL131	ZSU234	Classification Accuracy (%)
**Baseline**	72.96	78.57	89.80	81.54	**99.64**	86.13	95.62	96.70	98.54	**97.08**	90.81
**TPLBP**	61.73	77.04	85.20	78.97	92.34	**97.81**	98.54	97.43	98.54	88.32	89.19
**Fused**	**88.27**	**90.81**	**90.31**	**90.26**	98.18	97.08	**98.54**	**98.17**	**99.27**	**97.08**	**95.43**

**Table 4:** The classification accuracy of different features.

Features	Baseline	TPLBP	Cascaded	Fused
**Classification accuracy (%)**	85.70	83.80	91.01	**93.61**

**Table 5:** The classification accuracy of different algorithms.

Algorithms	SVM [36]	SRC [36]	CNN [37]	SAE	Proposed Method
**Classification accuracy (%)**	86.73	89.76	92.30	93.61	**95.43**

**Table 6:** The 10-class targets confusion matrices of two methods.

**SAE trained on-images**		**BMP2**	**BTR70**	**T72**	**BTR60**	**2S1**	**BRDM2**	**D7**	**T62**	**ZIL131**	**ZSU234**
	**BMP2**	88.3	2.1	0.5	0.5	6.1	0.5	1.0	1.0	0.0	0.0
	**BTR70**	0.5	**92.9**	0.0	0.5	5.6	0.0	0.0	0.0	0.5	0.0
	**T72**	0.5	0.5	90.3	1.0	3.7	0.0	0.5	2.0	1.0	0.5
	**BTR60**	1.5	4.2	0.5	87.7	5.1	0.0	0.5	0.5	0.0	0.0
	**2S1**	0.0	1.8	0.4	0.0	90.5	1.8	0.4	0.7	4.4	0.0
	**BRDM2**	1.0	0.4	0.4	0.4	0.0	96.7	0.7	0.0	0.0	0.4
	**D7**	0.0	0.0	0.0	0.4	0.0	0.7	98.5	0.4	0.0	0.0
	**T62**	0.0	0.4	0.4	0.4	0.4	0.7	0.7	90.5	0.4	6.1
	**ZIL131**	0.0	0.0	0.0	0.0	0.0	0.0	0.4	0.4	99.2	0.0
	**ZSU234**	0.0	0.0	0.0	0.0	0.0	0.0	1.8	1.1	0.4	96.7
**The proposed method**		**BMP2**	**BTR70**	**T72**	**BTR60**	**2S1**	**BRDM2**	**D7**	**T62**	**ZIL131**	**ZSU234**
	**BMP2**	**88.3**	1.5	4.1	4.6	0.0	1.0	0.0	0.0	0.0	0.5
	**BTR70**	1.0	90.8	0.5	7.2	0.0	0.5	0.0	0.0	0.0	0.0
	**T72**	7.7	0.5	**90.3**	1.5	0.0	0.0	0.0	0.0	0.0	0.0
	**BTR60**	1.0	5.1	0.5	**90.3**	0.5	2.6	0.0	0.0	0.0	0.0
	**2S1**	0.0	0.0	0.0	0.0	**98.2**	0.0	0.3	1.5	0.0	0.0
	**BRDM2**	2.1	0.4	0.0	0.4	0.0	**97.1**	0.0	0.0	0.0	0.0
	**D7**	0.0	0.0	0.0	0.0	0.0	0.0	**98.5**	0.4	0.7	0.4
	**T62**	0.0	0.0	0.0	0.0	0.0	0.0	0.7	**98.2**	1.1	0.0
	**ZIL131**	0.0	0.0	0.0	0.0	0.0	0.0	0.4	0.4	**99.2**	0.0
	**ZSU234**	0.0	0.0	0.0	0.0	0.4	0.0	1.8	0.7	0.0	**97.1**

**Table 7:** Training time and testing time of different methods.

Consumed Time	Training Time (s)	Testing Time (s)
**SAE trained on images**	4254.53	3.19
**Proposed method**	340.69	0.044
